# *Streptomyces* sp. Strain MUSC 125 from Mangrove Soil in Malaysia with Anti-MRSA, Anti-Biofilm and Antioxidant Activities

**DOI:** 10.3390/molecules25153545

**Published:** 2020-08-03

**Authors:** Hefa Mangzira Kemung, Loh Teng-Hern Tan, Kok-Gan Chan, Hooi-Leng Ser, Jodi Woan-Fei Law, Learn-Han Lee, Bey-Hing Goh

**Affiliations:** 1 Biofunctional Molecule Exploratory Research Group (BMEX), School of Pharmacy, Monash University Malaysia, Bandar Sunway 47500, Selangor Darul Ehsan, Malaysia; hefa.kemung@monash.edu; 2 Novel Bacteria and Drug Discovery Research Group (NBDD), Microbiome and Bioresource Research Strength (MBRS), Jeffrey Cheah School of Medicine and Health Sciences, Monash University Malaysia, Bandar Sunway 47500, Selangor Darul Ehsan, Malaysia; loh.teng.hern@monash.edu (L.T.-H.T.); hooileng_ser@y7mail.com (H.-L.S.); jodi.law1@monash.edu (J.W.-F.L.); 3 Division of Genetics and Molecular Biology, Institute of Biological Sciences, Faculty of Science, University of Malaya, Kuala Lumpur 50603, Malaysia; 4 International Genome Centre, Jiangsu University, Zhenjiang 212013, China; 5 College of Pharmaceutical Sciences, Zhejiang University, Hangzhou 310058, China; 6 Health and Well-Being Cluster, Global Asia in the 21st Century (GA21) Platform, Monash University Malaysia, Bandar Sunway 47500, Subang Jaya, Malaysia

**Keywords:** *Streptomyces*, mangrove, anti-MRSA, anti-biofilm, antioxidant, GC-MS, PKS

## Abstract

There is an urgent need to search for new antibiotics to counter the growing number of antibiotic-resistant bacterial strains, one of which is methicillin-resistant *Staphylococcus aureus* (MRSA). Herein, we report a *Streptomyces* sp. strain MUSC 125 from mangrove soil in Malaysia which was identified using 16S rRNA phylogenetic and phenotypic analysis. The methanolic extract of strain MUSC 125 showed anti-MRSA, anti-biofilm and antioxidant activities. Strain MUSC 125 was further screened for the presence of secondary metabolite biosynthetic genes. Our results indicated that both polyketide synthase (*pks*) gene clusters, *pks*I and *pks*II, were detected in strain MUSC 125 by PCR amplification. In addition, gas chromatography-mass spectroscopy (GC-MS) detected the presence of different chemicals in the methanolic extract. Based on the GC-MS analysis, eight known compounds were detected suggesting their contribution towards the anti-MRSA and anti-biofilm activities observed. Overall, the study bolsters the potential of strain MUSC 125 as a promising source of anti-MRSA and antibiofilm compounds and warrants further investigation.

## 1. Introduction

The emergence of antibiotic-resistant bacteria poses a serious global health problem in which antibiotic-susceptible pathogenic bacteria mount defensive mechanisms against effects of current antibiotics [1]. It is worrisome, as these antibiotic-resistant bacteria have also been identified in food sources [2]. Methicillin-resistant *Staphylococcus aureus* (MRSA) is a strain of the Gram-positive bacteria *Staphylococcus aureus* that has acquired resistance towards methicillin, just one year after its introduction in 1960 [3]. Over the years, MRSA has grown resistant to other classes of antibiotics [4]. To date, MRSA constitutes one of the major impediment to treatment strategies of infectious diseases, particularly in hospital settings, affecting mostly immunocompromised patients [5] with a high prevalence of MRSA (<50%) reported in the United States, South America and Asia [6]. It has been known that MRSA cause severe complications, such as pneumonia and even sepsis, in which if not treated immediately, can progress on to death. The persistence and spread of MRSA in hospital settings is of great concern, as MRSA has been shown to produce protective biofilm layers [7] rendering antibiotics less effective in eradicating them. In spite of efforts to introduce several new anti-MRSA drugs, which are derivatives of traditional antibiotics [6], vancomycin and daptomycin remain superior in treating complicated MRSA infection [8]. Yet, few studies from clinical settings reported findings of MRSA from patients treated with vancomycin [9] and daptomycin [10]. The limited treatment options at hand coupled with the growing resistance to the last resort first-line anti-MRSA drugs has urged the World Health Organization in early 2017 to consider MRSA in the list of antibiotic-resistant bacteria for new drug development [11]. 

The Gram-positive bacteria *Streptomyces* is a genus belongs to Actinobacteria phylum and exhibits mycelial growth characteristics [12]. The genus *Streptomyces* continues to be an important microbe for research as evidenced having the largest number of validly published species with 874 species recorded in the database of List of Prokaryotic names with Standing in Nomenclature (LPSN) [13] at the time of writing (24/4/2020). Moreover, it remains the most sought after microbe for production of biologically active metabolites [14,15]. Many of the antibiotics routinely used for treating infections caused by bacteria have been naturally recovered from *Streptomyces* [16] including the anti-MRSA drugs vancomycin and daptomycin that are used to treat severe infections associated with MRSA. According to a review by Kemung, et al. [17], *Streptomyces* have proven to be a great source of anti-MRSA and anti-biofilm compounds. In fact, more than 124 compounds showing anti-MRSA activity have been previously reported from *Streptomyces*. They remain to date, a valuable resource of new drugs with renewed interest in *Streptomyces* from mangrove forest ecosystems [18]. 

Mangrove forests are an unique ecosystem situated between the coastal margins and marine ecosystems and are located in 118 coastal and sub-tropical countries [19]. Given their unique geographical location, mangrove forests and other living organisms, therein, have adapted to grow exclusively in warm tropical and subtropical climates (>24 °C) [20] while constantly exposed to the harsh saline ocean waters as well as changing tides and acidic soil [21]. These circumstances are driving the living organisms thriving in mangrove ecosystem to evolve and to withstand these harsh environmental conditions. Hence, mangrove ecosystems are regarded as a “productive” ecosystem whereby the residing microorganisms develop physiological functions that enable the production of enzymes and molecules to cope with the harsh conditions [22]. A rich biodiversity translates to a rich chemical diversity, and this is evidenced in that various resources are derived from mangroves and utilized in traditional medicines [23]. The microbiological diversity in the mangrove soil, particularly the *Streptomyces*, and their biosynthetic potentials remain underexplored [24]. Many of the lead compounds reported from mangrove forests were isolated from endophytic fungi with few reported from *Streptomyces* [25]. 

*Streptomyces* are aerobic saprophytes and feed on dead and decaying materials [12] by the production of various extracellular enzymes [26]. Although *Streptomyces* are predominantly aerobic bacteria, they thrive in an anoxic mangrove soil environment where they are constantly exposed to oxidative stress. It is not surprising to find *Streptomyces* from stressful ecosystem such as mangrove forest, produces secondary metabolites to assist in the defence mechanisms, particularly antioxidants are used to prevent oxidative damage. The antioxidant potential of several mangrove *Streptomyces* in Malaysia have been reported [27,28,29]. To date, the mangrove forest remains largely an understudied ecosystem with respect to investigating their secondary metabolites which are valuable sources for future development of therapeutics [24]. The aim of the current study was to evaluate the anti-MRSA, anti-adherence and antioxidant potentials of a mangrove-derived *Streptomyces* sp. strain MUSC 125. The study revealed that strain MUSC 125 methanolic extract exhibited anti-MRSA, anti-adherence property towards the MRSA biofilm formation and antioxidant activity. The gas chromatography-mass spectroscopy (GC-MS) detected eight known compounds suggesting their possible involvement in the anti-MRSA, anti-adherence, and antioxidant activities. 

## 2. Results

### 2.1. Phylogenetic Analysis of Strain MUSC 125

**Figure 1:**
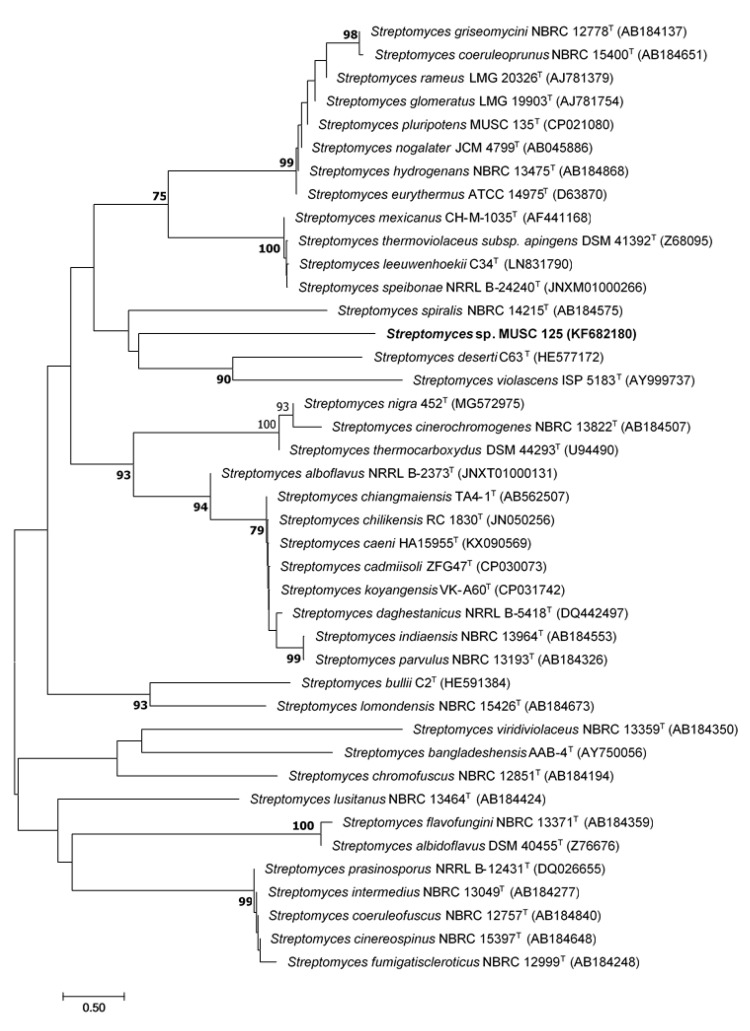
Neighbour-joining phylogenetic tree based on 1491 nucleotides of the 16S rRNA gene sequence showing the relationship between strain MUSC 125 and representatives of related taxa. Numbers and nodes indicate percentages (>50%) of 1000 bootstrap re-sampling. Bar, 0.5 substitutions per site.

The partially complete 16S rRNA gene sequence of strain MUSC 125 strain (GenBank accession number KF682180) was aligned with other strains that appeared to share highly similar 16S rRNA gene sequences from GenBank/EMBL/DDBJ database. Strain MUSC 125 showed the highest percentage of 16S rRNA gene sequence similarities of 99.93% to *Streptomyces pluripotens* MUSC 135^T^ and followed by *Streptomyces cinereospinus* (99.24%) and *Streptomyces mexicanus* (99.17%). The phylogenetic tree constructed showed strain MUSC 125 forming distinct clade with *Streptomyces deserti* C63^T^ and *Streptomyces violascens* ISP5183^T^ (Figure 1). The culture was deposited in the Marine Culture Collection of China, Third Institute of Oceanography, State Oceanic Administration, Fujian (P.R. China), and the accorded accession number is MCCC 1K03220. 

### 2.2. Phenotypic Characterization of Strain MUSC 125

The strain MUSC 125 grew well on International Streptomyces Project (ISP) 2, ISP5, ISP6, ISP7 and SCA and NA after 1–2 weeks at 28 °C (Supplementary Materials Table S1). The cell morphology, as illustrated in Figure S1, showed rich thread-like growth with no visible sign of fragmentation at 1–2 weeks of growth on ISP 2 media. This observation is common among strains of *Streptomyces* genus [30]. The growth of colonies was observed on all the different culture media used in the experiment, except on ISP4. Soluble pigments were produced when grown on ISP6 and NA as dark olive brown and greyish greenish yellow, respectively. Growth was observed at optimal temperatures of 32–36 °C, an optimal pH of 7 and optimal NaCl concentrations of 0–2%. The biochemical tests that were applied demonstrated the potential of strain MUSC 125 to produce catalase while also hydrolyse starch and tributyrin (Table S2). 

### 2.3. Anti-MRSA Activity by Agar Well Diffusion Assay 

To determine whether an extract exhibits anti-MRSA activity, both the agar well diffusion assay and MIC test were performed according to the guidelines of Clinical Laboratory Standards Institute (CLSI). By following the CLSI guideline for a controlled condition, such as incubation temperature, duration of incubation of test bacteria, colony forming unit (CFU/mL), and type of culture medium, are important factors which allow for proper comparison of anti-MRSA activity with the reference strain and test drugs and ultimately determine whether an extract has anti-MRSA activity [31,32]. The agar-well susceptibility test is based on the principle of allowing test samples dispensed into the pre-dug wells to diffuse through the agar for up to 24 h. Test bacteria that are susceptible to the test sample at given concentrations tend to produce a clear halo zone around the agar well. The anti-MRSA activity is determined by the size (in mm) of the cleared ZOI; the greater the zone of inhibition in diameter, the greater the anti-MRSA activity [32,33,34]. The preliminary anti-MRSA test result was sufficient to demonstrate the anti-MRSA property of methanolic extract MUSC 125 towards MRSA ATCC 43300 showing a ZOI of 19 ± 0 mm, while the ZOI measured 19.33 ± 0.58 mm against MRSA ATCC 33591 (Table S3 and Figure S2). The ZOI exhibited by the positive control vancomycin disc at 30 µg was 15 ± 0 mm against MRSA ATCC 43300 and 15.3 3 ± 0.47 mm against MRSA ATCC 33591 (Table S3 and Figure S2) and demonstrated the reliability of the anti-MRSA agar-well diffusion assay.

### 2.4. Minimum Inhibitory Concentration (MIC) and Minimum Bactericidal Concentration (MBC) of Methanolic Extract MUSC 125

Since the agar-well susceptibility test does not quantify the inhibitory power of test samples, it is often accompanied by a MIC test [35]. Moreover, the supplementary data by Kemung, et al. [17] supports this, showing anti-MRSA studies of *Streptomyces* initially employ a preliminary test, such as an agar-well susceptibility test, followed by MIC. Here, the MIC was performed in a 96 well plate which facilitated a wider sample concentration range and reduced the overall volume of test samples. The MIC was determined by a clear well without any turbidity relative to control growth with >90% inhibition of bacterial growth [31]. The results from the MIC test of MUSC 125 were 12.5 mg/mL against MRSA ATCC 43300 and 25 mg/mL against MRSA ATCC 33591, respectively. The MIC of vancomycin against MRSA ATCC 43300 and MRSA ATCC 33591 were 5 µg/mL and 10 µg/mL, respectively. The MBC could not be established since it was ≥50 mg/mL (Table S4).

### 2.5. Anti-Biofilm/Anti-Adherence Activity of Methanolic Extract of Strain MUSC 125

The anti-biofilm potential of methanolic extract MUSC125 was investigated by the use of the traditional crystal violet staining method. The crystal violet would stain biofilm biomass, facilitating colorimetric detection of its presence. Hence, the dark purple colour after addition of crystal violet confers colorimetric evidence for the presence of biofilm (Figure S3A). The anti-biofilm activity was further quantified in terms of the absorbance of crystal violet solution in ethanol at 570 nm. The absorbance reading of the concentration of crystal violet is an indirect representation of the total biofilm biomass per extract concentration tested. Hence, the anti-biofilm activity of methanolic extract of MUSC 125 showed a concentration-dependent activity with the clear solution at 1.5625 mg/mL (Figure S3B). The anti-biofilm potential of the methanolic extract MUSC 125 was evaluated using the anti-adherence assay. The result of the assay showed inhibition of the biofilm formation at 1.5625 mg/mL. In general, the methanolic extract demonstrated anti-biofilm potential at 1/8× MIC of methanolic extract (Figure S3B). The wells observed with the naked eye also indicated the anti-biofilm potential of the methanolic extract MUSC 125 (Figure S3A).

### 2.6. Antioxidant Assays

#### 2.6.1. 2,2’-azino-bis(3-ethylbenzothiazoline-6-sulfonic acid) (ABTS) and 2,2-diphenyl-1-picrylhydrazyl (DPPH) Antioxidant Assays

The present work investigated the potential of methanolic extract MUSC 125 showing an overall strong antioxidant activity when screened against four antioxidant assays. Together, the ABTS and DPPH radical scavenging assays proved to be effective in establishing the in vitro antioxidant potential of extracts and are often included in many in vitro antioxidant studies [36]. Both ABTS and DPPH are free radicals seeking electrons from electron-rich compounds such as phenolic compounds. Given that methanolic extract MUSC 125 possesses electron-rich constituents, a reaction would occur involving transfer of electrons to neutralize free radicals. The free radicals that remain after the reaction are detected by the UV light and their absorbance calculated as a percentage scavenging activity (%). Collectively, the ABTS and DPPH scavenging activity recorded for methanolic extract of MUSC 125 were 67.85% ± 2.16% for ABTS and 64.77% ± 1.31% for DPPH at a highest concentration of 4 mg/mL (Table S5). This suggested that extract MUSC 125 had effectively scavenged the majority of the free ABTS and DPPH radicals. 

#### 2.6.2. Metal Chelating Assay 

The metal chelating potential of *Streptomyces* sp, strain was briefly mentioned by Ser, et al. [37] without presenting the detailed analysis. Herein, the metal chelating activity of strain MUSC 125 was investigated by assessing how well the extract chelate the ferrous ions (Fe^2+^) via a ferrozine competition assay. A decrease in the Fe^2+^–ferrozine complex is an indication that the extract had competitively formed a complex with Fe^2+^ [38]. In this experiment, there was a notable decrease in the absorbance reading, suggesting a lower concentration of a ferrous ion–ferrozine complex. The metal chelating activity of methanolic extract MUSC 125 was recorded at 52.75% ± 1.76% and 61.10% ± 0.64% at 2 mg/mL and 4 mg/mL, respectively (Table 1).

#### 2.6.3. Ferric Reduction Antioxidant Power (FRAP) Assay

In addition to ABTS, DPPH and metal chelating antioxidant methods, the FRAP antioxidant assay was also conducted. In the FRAP experiment, the Fe^3+^ and the methanolic extract MUSC 125 participate in a redox reaction which resulted in the Fe^3+^ being reduced to Fe^2+^ [29]. The reduced potassium ferrous cyanide was afterwards allowed to react with ferric chloride forming a dark blue complex which has an absorbance wavelength at about 700 nm [29]. Based on the current FRAP experiment, the absorbance (*p* < 0.05) of methanolic extract MUSC 125 ranged from 1.53 ± 0.06 to 1.55 ± 0.05 in the dose range of 1–2 mg which was equivalent to 0.22–0.23 µg of ascorbic acid (Figure S4).

### 2.7. Total Phenolic Content (TPC) Determination with Folin–Ciocalteu’s Reagent Method

The TPC test using Folin–Ciocalteu’s reagent was performed to determine the presence of phenolic compounds in the methanolic extract MUSC 125. In the presence of a reducing agent, the yellow Folin–Ciocalteu’s reagent will be reduced, indicated by the change in colour of the solution to dark blue [29]. The visible colour change suggested the presence of phenolic compounds with the strongest blue colour at 4 mg/mL of the methanolic extract MUSC 125. According to the Pearson’s correlation analysis, there was a strong relationship between the phenolic constituents in methanolic extract MUSC 125 and antioxidant tests revealing an R value of 0.998 (ABTS), 0.942 (DPPH) and 0.974 (metal chelating) which altogether suggests that the presence of phenolic content may have assisted in the antioxidant activity of the methanolic extract MUSC 125 (Table S5).

### 2.8. Detection of the Polyketide Synthase (pks) and Non-Ribosomal Peptides Synthase (nrps) Genes in Strain MUSC 125

**Figure 2:**
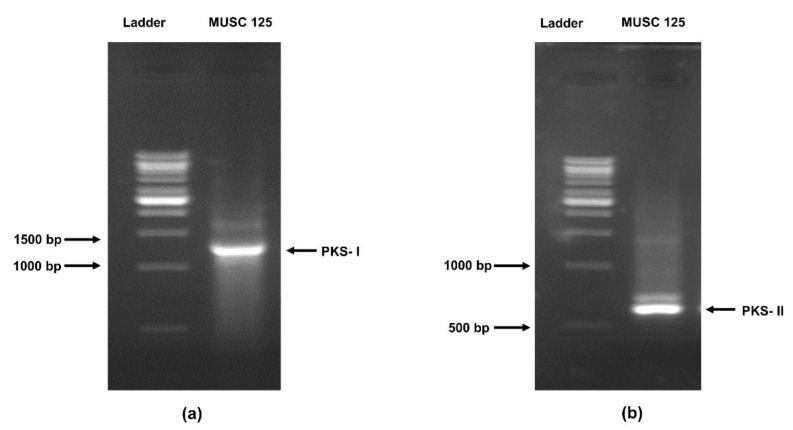
Detection of PCR products in the strain MUSC 125 on agarose gel: (**a**) PKS I (1200–1400 bp) and (**b**) PKS II (600 bp). PKS — Polyketide synthase.

The genus *Streptomyces* has been identified to possess an average of 20 biosynthetic gene clusters [39,40,41]. Much of secondary metabolites microbes produce are PKS and NRPS and constitute an estimated 50–75% of biosynthetic gene clusters for secondary metabolites. According to a literature review conducted recently citing references from inception to 2018, it was found that out of the 124 anti-MRSA compounds isolated from *Streptomyces*, 53 were polyketides followed by NRPS and others [17]. It is not surprising that both *pks*I (band size 1200–1400 bp) and *pks*II (band size 600 bp) are present in strain MUSC 125 (Figure 2). Further, BLAST analysis of the *pks*I gene cluster from strain MUSC 125 showed the closest resemblance to that of *Streptomyces pluripotens* strain MUSC 137^T^ and MUSC 135^T^ at 99.32% gene sequence similarity. *Streptomyces pluripotens* MUSC 135^T^ previously showed broad-spectrum antibacterial activity including anti-MRSA activity and antioxidant potential [42], whereas *Streptomyces pluripotens* MUSC 137^T^ exhibited antioxidant and anticancer properties [43].

### 2.9. Gas Chromatography-Mass Spectrometry (GC-MS) Analysis of Methanolic Extract of Strain MUSC 125

**Figure 3:**
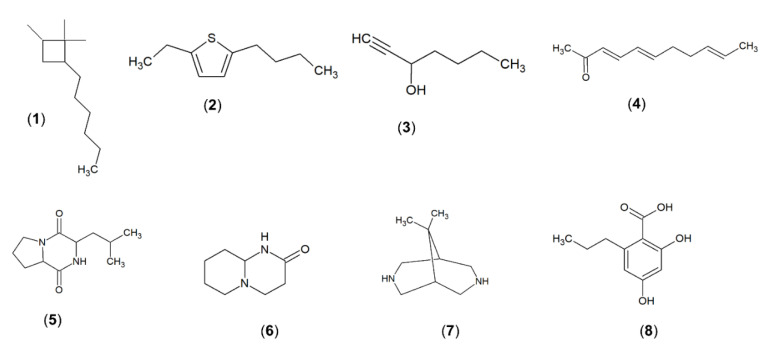
Chemical structures of compounds detected in the methanolic extract strain MUSC 125.

The promising results of antioxidant and anti-MRSA activities have prompted further investigation to analyse the chemical compounds present in the methanolic extract MUSC 125. Previous studies have utilized GC-MS to perform the chemical analysis for the putative identification of chemical compounds in microbial extracts after determining their biological activities [38,44,45,46]. The GC-MS analysis resulted in eight known compounds detected in the methanolic extract MUSC 125 (Table 2 and Figure 3): cyclobutane,2-hexyl-1,1,4-trimethyl (**1**); thiophene, 2-butyl-5-ethyl **(2)**; 1-heptyn-3-ol (**3**); 8-[*N*-aziridylethylamino]-2-6,dimethyloctene-2 **(4)**; pyrrolo[1,2-a]pyrazine-1,4-dion,hexahydro (**5**); octahydro-2H-pyrido(1,2-a)pyrimidin-2-one (**6**); 9,9-dimethyl-3,7-diazabicyclo[3.3.1]nonane, **(7)**; and 2,4-dihydroxy-6-propylbenzoic acid (**8**).

## 3. Discussion

In recent years, the search for *Streptomyces* is concentrated on the rather less explored ecological niches such as the mangrove forest to increase chances of finding new *Streptomyces* species with bioactive potentials [24,56,57]. An earlier study by Ser, et al. [37] demonstrated the large genome size of *Streptomyces* sp. strain MUSC 125 reported to have antioxidant activity. Large genome size (~7.66 Mbp) and high G+C content (70%) of strain MUSC 125 showed genes associated with secondary metabolisms, iron acquisition and metabolism as well as amino acids and derivatives among others. Additionally, Ser, et al. [37] reported that MUSC 125 had antioxidant potential (metal-chelating) and it is well supported by the findings of this study based on the multiple antioxidant assays tested on the methanolic extract of MUSC 125.

To date, there are a limited number of studies conducted that have investigated the anti-MRSA potential of mangrove-derived *Streptomyces* in Malaysia [17]. Nevertheless, an interesting study of a mangrove *Streptomyces* in Malaysia revealed the discovery of the novel *Streptomyces pluripotens* sp. nov. exhibiting anti-MRSA activity [58]. Additional studies are therefore needed to increase the chances of discovering *Streptomyces* exhibiting anti-MRSA activity. The current study of strain MUSC 125 was undertaken specifically for this purpose. The phylogenetic analysis indicated that strain MUSC 125 is well positioned within the *Streptomyces* genus (Figure 1). Interestingly, even though both strain MUSC 125 and *Streptomyces pluripotens* MUSC 135^T^ have relatively high percentages of 16S rRNA sequence similarity (99.93%), strain MUSC 125 is closely related to *Streptomyces deserti* C63^T^ based on their evolutionary distance from the phylogenetic analysis. This result suggests that strain MUSC 125 is tentatively a different bacterial species from *Streptomyces pluripotens* MUSC 135^T^, and they may exhibit differential bioactive potentials. *Streptomyces* are generally described as heterogenous, even at the strain level in terms of their metabolic profiles [59,60]. Waksman, et al. [59] examined different strains of the *Streptomyces griseus* and found that only a few were able to produce streptomycin as the major product and this was in addition to producing an antifungal actidione. Other non-streptomycin-producing *Streptomyces griseus* yielded grisein as the major antibiotic product [61]. Moreover, cultivation of *Streptomyce*s should consider each strain as different and may respond differently to fermentation condition applied. Hence, yielding a product that is either different or not desired [62].

Moreover, this study further examined the phenotypic characteristics of strain MUSC 125 systematically to have a better understanding of the strain in terms of its biochemical and physiological properties. The image of strain MUSC 125 captured under the scanning electron microscope (SEM) showed morphological features consistent with the *Streptomyces* genus (Figure S1). Moreover, strain MUSC 125 grew on all culture media selective for promoting growth and identification of *Streptomyces* with the exception of ISP4 (Table S1). The observation of colony colours on both the vegetative and aerial growth point to characteristics of *Streptomyces*. Additionally, soluble pigments diffused noticeably through ISP 6 and NA culture media (Table S1).

Microbes in mangrove soil have adapted to the soil acidity, salinity, temperature and constant tidal changes [63]. This was evident for strain MUSC 125, as growth characteristics indicated physiological tolerance to growth in the temperature range of 18–40 °C, acidity tolerance of pH 4–7 and salinity levels up to 0–6 (%) (Table S2). These values fall within the range tolerated by microbes in the mangrove of Pahang, Malaysia [26,29]. Microbial resilience is a fundamental factor driving the production of secondary metabolites necessary for survival in an oxygen-deprived environment, such as the mangrove soil. In recent years, secondary metabolites produced by microbes residing in the soil of mangrove forest, have been explored through careful investigation of the in vitro antibacterial and antioxidant potentials [28]. *Streptomyces* deserve greater attention for antioxidant studies of their secondary metabolites, cognizant of the fact, that prior studies of few have shown promising results [26,29].

It is widely known that phenolic compounds exhibit a number of biological activities including antioxidant and antibacterial [64,65]. There is also evidence demonstrating that antioxidative compounds exhibit promising anti-MRSA activity [66,67,68]. In this study, we investigated the presence of phenolic compounds in the methanolic extract MUSC 125. It is known that the antioxidant activities of phenolic compounds are mediated by the hydroxyl functional group that donate their electrons through hydrogen transfer. Phenolic compounds are polar permitting and can be efficiently extracted using solvents that can extract polar compounds. A study by Noreen, et al. [69] showed total phenolic content was 15 fold higher in ethanol and less in dichloromethane. Furthermore, ethanolic extract showed the strongest antioxidant activity against DPPH and ABTS free radicals. Moreover, a study by Johari and Khong [70] demonstrated that methanolic extract demonstrated highest TPC followed by chloroform and lastly hexane which indicate the decline in polarity level of the solvents. The antioxidant activity (DPPH) was strongest in methanolic extract compared to the other to extract. The antibacterial activity of these three extracts were measured and the MIC (225 µg/mL) were higher against *Staphylococcus aureus* and *Streptococcus pyogenes* compared to hexane (1800 µg/mL) [70]. Nevertheless, there are exceptions where non-polar extracts showed the strongest antioxidant activity as demonstrated by Prabhakar, et al. [71].

As a phenolic compound detected in the methanolic extract MUSC 125, compound (**8**), which is also known as divaric acid, was first reported from lichen in 1985 [72]. Interestingly, compound (**8**) was detected in hexane extract of a lichen which was shown to have antibacterial activity on *Staphylococcus aureus*, *Bacillus subtilis* and *Streptococcus mutans* in addition to DPPH antioxidant activity [54]. In another study, compound (**8**) was detected in the methanolic extract of *Evernia divaricate* exhibiting antibacterial activity against some Gram-positive and Gram-negative bacteria including *S. aureus* [55]. The *ortho* position of hydroxyl group was suggested to be associated with stronger scavenging activity [73]. Even though there are so far no report on the anti-MRSA mechanism of action of compound (**8**), a study previously reported investigating the antimicrobial activity of phenolic compounds in wild mushrooms utilizing a structure–activity relationship and docking models in order to determine the mechanism of action of active compounds. In terms of the structure–activity relationship, it was found that phenolic compounds 2,4 dihydroxybenzoic acid, vanillic, syringic and *p*-coumaric selectively inhibited MRSA but was not susceptible to *S. aureus* [74]. Furthermore, the study revealed that the presence of functional groups COOH, two hydroxyl groups in *para* and *ortho* position of the benzene ring, a methoxyl group in the *meta* position as necessary for eliciting anti-MRSA activities. Moreover, docking studies showed that 2,4-dihydroxybenzoic acid, vanillic acid and syringic acid were superimposed on PBP2A protein in MRSA [74]. Since compound (**8**) is 2,4-dihydroxy-6-propyl-benzoic acid, it may be likely that the two hydroxyl groups and the carboxylic acid may be responsible for the observed anti-MRSA effect. Yet, further studies on compound (**8**) are required to fully understand its mechanism of action on MRSA. A couple of studies have observed cell content leakage caused by interaction of phenolic compounds with the structures of cytoplasmic membrane [75] whilst potentiation of antibacterial activity was observed when used in conjunction with antibiotics [76].

In terms of the anti-biofilm activity, one of an iron chelating dihydrobenzoic acid, namely, 2,3-dihydroxybenzoic acid was shown to significantly reduce biofilm formation against one of the MRSA strains tested. In addition, it was revealed that the presence of ferric chloride was required for biofilm formation by the MRSA. The combined use of nisin with 2, 3-dihydroxybenzoic acid reduced biofilm formation by 88% compared to use of nisin alone or 2, 3-dihydroxybenzoic acid alone with reduction of only 3% and 63%, respectively [77]. In general, anti-biofilm activity of phenolic compounds may be through inhibiting bacterial virulence factors such enzymes and toxins [78]. Further studies, however, are required to elucidate the anti-biofilm action of compound (**8**).

Vancomycin is the standard drug for treatment for severe MRSA blood stream infection. Vancomycin is a glycopeptide and has a relatively large structure, making it difficult to penetrate into the pre-formed biofilm [79]. Despite that, vancomycin has been often used as positive control in previous antibiofilm studies. Specifically, vancomycin was used to prevent biofilm formation, whereby it was shown to exert antibiofilm effect by inhibiting the formation of biofilms as evidence, in studies by Rane, et al. [80] and Beeton, et al. [81]. Similarly, vancomycin was used as the positive control to ensure the reproducibility and reliability of the results obtained from the antibiofilm experiment of this study. The antibiofilm experiment demonstrated that the methanolic extract MUSC 125 was effective to inhibit biofilm formation by MRSA at 1/8 × MIC (1.5625 mg/mL) which was the lowest concentration tested.

Thus far, *Streptomyces pluripotens* MUSC 135^T^ is the only reported *Streptomyces* strain isolated from mangrove Malaysia showing anti-MRSA activity in well diffusion assay [58]. Although methanolic extract of MUSC 125 exhibited anti-MRSA activity, the MIC values are relatively high when compared with other recent works [82,83], suggesting that the active compound that targets MRSA could be present in the complex mixture of crude extract at a relatively low concentration. It is known that the cultivation conditions greatly influence the secondary metabolites production in *Streptomyces*. Perhaps, future investigation could focus on the optimization of culture conditions to enhance the antibacterial efficacy of strain MUSC 125. Moreover, fractionation of the crude extract with the bioassay guided approach is an effective strategy to improve the overall activity of the extract.

Majority of the *Streptomyces* anti-MRSA compounds from numerous ecological niches are polyketides [17]. Polyketides refer to large group of microbial secondary metabolites characteristic of multiple ketides [84]. Interestingly, some of the clinically useful drugs derived from *Streptomyces* are macrocyclic polyketides (eg. Erythromycin), or aromatic polyketides (e.g. tetracycline, doxorubicin) [85]. Macrocyclic polyketides and aromatic polyketides are biosynthesized by multifunctional enzymes. The primary enzyme for biosynthesis of macrocyclic polyketids are polyketide synthase type 1 (PKS I) whereas PKS II is responsible for synthesis of aromatic polyketides [85]. The purpose for carrying out this experiment was to investigate the presence of the biosynthetic gene cluster *pks* I and *pks* II in the genome of strain MUSC 125, thereby to predict the biosynthetic potential of strain MUSC 125 in the production of polyketides which may exhibit anti-MRSA or anti-biofilm activities. The findings obtained from the experiment indicated the presence of PKS I and PKS II in strain MUSC 125. This result was important, as it suggests the potential of strain MUSC 125 to produce cryptic polyketides which may have potential in becoming useful therapeutic agents.

Among the detected compounds by GC-MS analysis, compound (**5**) has been the most frequently detected compound in microbial extracts including *Streptomyces*, demonstrating antioxidant and anti-*Staphylococcus aureus* activities [44,52]. Based on the literature search to date, there have been no reports on the underlying mechanism of action regarding anti-MRSA nor antioxidant activity from compound (**5**). Nevertheless, compound (**5**) was assumed to contribute to the antioxidant activity observed in the methanolic extract of the novel *Streptomyces mangrovisoli* sp. nov. [86]. In fact, one study reported the isolation and identification of compound (**5**) in ethyl acetate extract as active against multidrug-resistant *Staphylococcus aureus* clinical strains and scavenging DPPH free radicals. Furthermore, the isolated compound was non-cytotoxic towards mouse embryo fibroblast and non-haemolytic. Hence, compound (**5**) was recommended as an interesting compound that warrants further study considering its safety, non-haemolytic property as well as having dual role of reducing oxidative stress and inhibiting multidrug-resistant *Staphylococcus aureus* strains [87].

Other compounds detected by GC-MS included compound (**4**) which was previously reported in essential oil of few *Camellia* species to demonstrate activities against bacterial pathogens and ROS [50]. The essential oil of *Ballota saxatilis* containing compound (**4**) exhibited antioxidant activity as well as anticancer activity [51]. Antifungal and antioxidant activities were observed in an extract containing compound (**3**) [49]. Compound (**2**) was previously reported in the essential oil of *Linum pubescens* showing antibacterial activities against *S. aureus*, *E. faecalis*, *B. cereus, K. pneumoniae* and *E. coli*. A methanolic extract containing compound (**7**) from *Acinetobacter baumanii* demonstrated antifungal activity [52]. Compound (**1**), was detected in chloroform extract of leaf of *Datura stramonium* [47], while compound (**6**) was detected in the ethyl acetate extract obtained from the fungal endophyte *Colletotrichum gloeosporioide* in *Phlogacanthus thyrsiflorus Nees* [53]. Nevertheless, the GC-MS analysis provided insight into the composition of the potential compounds present in methanolic extract of MUSC 125 which may have responsibilities for the bioactivities. The multi-components crude extract may have conferred bioactivities as a result of a target compound or through synergism effects between two or more bioactive metabolites. However, the exact identities of the detected compounds can only be confirmed with further efforts by isolation and identification with the use of nuclear magnetic resonance or the comparing the chromatographic behaviour of the different components to that of authentic samples or commercial standards.

## 4. Materials and Methods 

### 4.1. Isolation Source and Maintenance of Strain MUSC 125

The soil samples for the present study were collected from mangrove forest in Tanjung Lumpur, Malaysia (MUSC-TLS4 3°48′21.3” N 103°20′3.3”E) in December 2012 [88]. The soil samples collected consisted of a segment of the soil layer just beneath 2–3 mm of the surface with a depth of 20 mm and was achieved with a sterile trowel. Soil samples were aseptically packed into sealable bags, delivered safely to be stored at −20 °C followed by air-drying. Air-dried samples were then ground and processed by wet-heat sterilization. Pre-treated samples were suspended in previously autoclaved water and diluted and plated uniformly across ISP 2 media supplemented with antifungal drugs which selectively promoted growth of *Streptomyces*. Growth was monitored by continuous sub-culture onto freshly made ISP 2 media until pure isolates was achieved. Pure isolates were then kept on ISP2 agar slant and 20% glycerol at −20 °C as stocks for future work.

### 4.2. Extraction of DNA and 16S rRNA Phylogenetic Analysis

The method of Hong, et al. [89] was used to perform genomic DNA (gDNA) extraction while amplification of 16S rRNA gene was based on the method of Lee, et al. [58]. In short, PCR reactions was run in a final volume of 50 μL on Kyratec PCR Supercycler (Kyratec, Queensland, Australia) according to the manufacturer’s protocol (Solgent^TM^, Daejeon, Korea) with cycling conditions: (i) 95 °C for 5 min; (ii) 35 cycles of 94 °C for 50 s, 55 °C for 1 min, and 72 °C for 1 min and 30 s; (iii) 72 °C for 8 min. The 16S rRNA gene sequence of strain MUSC 125 was compared and aligned with closely related strains accessed through GenBank/EMBL/DDBJ databases using the CLUSTAL-x software [90]. Phylogenetic tree based on neighbour-joining method was constructed [91] with MEGA version 6.0 software tool [92]. Evolutionary distances was computed based on Kimura’s two-parameter model for the neighbour-joining algorithm [93]. The EzBiocloud database was used to assess the 16S rRNA sequence similarities between strain MUSC 125 and the type strains [94]. Phylogenetic tree was also assessed for stability using Felsenstein bootstrap 1000 resampling method [95].

### 4.3. Phenotypic Characteristics of Strain MUSC 125

A 1–2-week old culture of strain MUSC 125 grown at 28 °C on several recommended media that promote *Streptomyces* growth for the purpose of characterizing its growth therein. The growth of strain MUSC 125 and whether it produced soluble pigment were assessed using the following media recommended in International *Streptomyces* Project (ISP), including ISP2, ISP3, ISP4, ISP5, ISP6, ISP7, *Streptomyces* agar (SA), Nutrient agar (NA), actinomycete isolation agar (AIA) and starch casein agar (SCA) [58]. The ISCC-BS standard colour chart was used to confer colony colour of strain MUSC 125 [96]. Strain MUSC 125 was also exposed to a temperature of 4 °C and rising to 50 °C, salinity levels of 0–10% and pH tolerance levels ranging from 2 to 10. The morphology of strain MUSC 125 at 1–2 weeks of growth on ISP 2 agar was viewed using a JEOL-JSM 6400 scanning electron microscope. To verify the presence of catalase, 3% (*v/v*) hydrogen peroxide was dropped onto strain MUSC 125 colony. Instant bubble formation indicated the presence of catalase [97]. The haemolytic activity of strain MUSC 125 was confirmed by the appearance of a clear zone around the 5 day old culture on blood agar [98]. Exoenzymes from strain MUSC 125 grown on ISP2 agar was detected using the method of Meena, et al. [99].

### 4.4. Fermentation and Extract Preparation of Strain MUSC 125

Seed culture of strain MUSC 125 was prepared aseptically in a small volume of 10 mL in tryptic soy broth (TSB) subjected to incubation for 10 days at 28 °C with an aeration rate of 200 rpm. Transfer of a 1 mL aliquot from the seed culture into freshly prepared 200 mL sterile Han’s Fermentation Media 1 (Biomerge, Selangor, Malaysia) and incubated under the aforementioned seed culture conditions. This was followed by centrifugation of the 10 day old culture broth at 4000 rpm at 4 °C for 5 min and then filtration to obtain the cell-free supernatant [58]. The supernatant was placed in a freeze-dryer at −45 °C for 3 days to remove water content. The organic solvent methanol was used to extract the secondary metabolites from the freeze-dried filtrate with subsequent filtration. The process was repeated three times. The resultant filtrate was further subjected to rotary vacuum evaporator for removal of methanol. Dried methanolic extract was stored kept at −20 °C for future work [45].

### 4.5. Anti-MRSA Activity of Methanolic Extract of Strain MUSC 125

Anti-MRSA assay was performed using agar well diffusion susceptibility test as described by CLSI with slight modification [32]. Colonies of MRSA strains (ATCC 43300 and ATCC 33591) were transferred aseptically to freshly prepared TSB (5 mL) and incubated at 37 °C, 220 rpm for 18–24 h. A bacterial suspension equivalent to 0.5 McFarland turbidity (1 × 10^8^ CFU/mL) was prepared. A hundred microliters aliquot of bacterial suspension was added onto Nutrient agar (NA) in a Petri dish and swabbed to produce uniform bacterial lawn. Wells with consistent sizes were dug with sterile Pasteur pipettes. The methanolic extract was prepared in ultrapure water containing 0.5% *v/v* dimethyl sulfoxide (DMSO). A hundred microliters aliquot (10 mg) of methanolic extract (100 mg/mL) were added into agar wells. Vancomycin 30 μg disc and DMSO (0.5% *v/v*) were used as positive and negative control, respectively. Petri dishes were subsequently sealed with parafilm and incubated at 37 °C for 18–24 h and the zone of inhibition (ZOI) expressed in millimetres (mm) was measured.

### 4.6. MIC and MBC of the Methanolic Extract MUSC 125

The MIC test was performed in a 96 well plate as described by Endo et al. [100] with slight modification. Briefly, a 50 µL of bacterial suspension (1 × 10^6^ CFU/mL) prepared in TSB media was loaded into each of the 96 well microplate except the negative control (TSB only). A series of 2 fold dilution was used to achieve final concentrations of crude extract ranging from 50 mg/mL, 25 mg/mL, 12.5 mg/mL, 6.5 mg/mL, 3.125 mg/mL to 1.5625 mg/mL in respective wells. Vancomycin, the untreated bacterial suspension and TSB alone, were used as the positive control, growth control and negative control, respectively. The 96 well plate was incubated at 37 °C for 24 h and the MIC determined afterwards. The MBC was determined by plating an aliquot of MIC, 2 × MIC and 4 × MIC cultures onto NA plates and incubated at 37 °C for 24 h. 

### 4.7. Anti-Biofilm/Anti-Adherence activity of the Methanolic Extract MUSC 125

The anti-biofilm test was conducted to determine the ability of extract to inhibit adhesion of MRSA cells onto the surfaces of the 96 well microtiter plate. The test was performed according to the method of Kemung, et al. [101]. A 50 µL (1 × 10^6^ CFU/mL) of a 24 hour bacterial culture was loaded into individual wells and followed by the additional of 50 µL constituted methanolic extract in the concentrations of 50 mg/mL, 25 mg/mL, 12.5 mg/mL, 6.25 mg/mL, 3.125 mg/mL and 1.5625 mg/mL. The plate was placed in the incubator for 24 h with temperature set at 37 °C. The wells were afterwards carefully emptied and washed thrice with sterile distilled water before being air dried. A 100 µL of aqueous crystal violet (0.1% *w/v*) was used to stain wells for 10 min. The wells were later rinsed with sterile distilled water 5 times and left to air dry. Ethanol (95 % *v/v*) was used to solubilize the crystal violet (CV) and absorbance of CV was measured at 570 nm. Percentage (%) attachment was calculated following Equation (1).

(1) $$\mathrm{Attachment}\;\left(\%\right)=\frac{\mathrm{Absorbance}\;\mathrm{of}\;\mathrm{sample}}{\mathrm{Absorbance}\;\mathrm{of}\;\mathrm{control}}\times \;100\;\%$$

### 4.8. Antioxidant Assays

#### 4.8.1. ABTS Scavenging Antioxidant Assay

The ABTS scavenging antioxidant assay was conducted to investigate ABTS scavenging potential of the methanolic extract MUSC 125. The ABTS antioxidant assay was performed according to Tan, et al. [38]. In short, ABTS radical (ABTS•^+^) was prepared from 7 mM of ABTS and 2.45 mM of potassium persulfate (K_2_S_2_O_8_). The six concentrations (0.125 mg/mL, 0.25 mg/mL, 0.5 mg/mL, 1 mg/mL, 2 mg/mL and 4 mg/mL) of methanolic extract MUSC 125 were prepared by 2 fold dilution in a 96 well plate. The ABTS radical was then dispensed into the 96 well plate. The plate was kept in the dark for 20 min prior to reading the UV absorbance at 734 nm. Gallic acid served as the standard for this experiment. Equation (2) was used to calculate the ABTS scavenging activity (%).

(2) $$\mathrm{ABTS}\;\mathrm{scavenging}\;\mathrm{activity}\;\left(\%\right)=\frac{\mathrm{Absorbance}\;\mathrm{of}\;\mathrm{control}-\mathrm{Absorbance}\;\mathrm{of}\;\mathrm{sample}\;}{\mathrm{Absorbance}\;\mathrm{of}\;\mathrm{control}}\times 100\%$$

#### 4.8.2. DPPH Scavenging Antioxidant Assay

Scavenging of DPPH radical by the methanolic extract was performed according to Tan, et al. [38]. The test was run on a 96 well microplate. A series of concentration of methanolic extract was prepared in the 96 well plate by 2 fold dilution ranging from 0.125 mg/mL, 0.25 mg/mL, 0.5 mg/mL, 1 mg/mL, 2 mg/mL to 4 mg/mL. A solution of DPPH ethanol (0.016% *w/v*) was then added into 96 well plates containing the methanolic extract and left standing in the dark for 20 min at room temperature. The UV absorbance of the mixture was read at a wavelength of 515 nm. Gallic acid was used as the positive control. Equation (3) was used to calculate the DPPH scavenging activity (%).

(3) $$\mathrm{DPPH}\;\mathrm{scavenging}\;\mathrm{activity}\;\left(\%\right)=\frac{\mathrm{Absorbance}\;\mathrm{of}\;\mathrm{control}-\mathrm{Absorbance}\;\mathrm{of}\;\mathrm{sample}}{\mathrm{Absorbance}\;\mathrm{of}\;\mathrm{control}}\times \;100\;\%$$

#### 4.8.3. Metal Chelating Assay

The metal chelating property of methanolic extract MUSC 125 was assessed through the metal chelating assay as described by Tan, et al. [38]. Methanolic extract MUSC 125 was prepared in the concentration of 0.125 mg/mL, 0.25 mg/mL, 0.5 mg/mL, 1 mg/mL, 2 mg/mL and 4 mg/mL and placed in individual wells. An addition of 2 mM ferrous sulphate (FeSO_4_) into the wells and followed by 5 mM of ferrozine. In this reaction mixture, both methanolic extract and ferrozine compete for ferrous ion (Fe^2+^). Ethylenediaminetetraacetic acid (EDTA) was employed as the standard in this experiment. Equation (4) was used to calculate the metal chelating activity (%).

(4) $$\mathrm{Metal}\;\mathrm{chelating}\;\mathrm{activity}\;\left(\%\right)=\frac{\mathrm{Absorbance}\;\mathrm{of}\;\mathrm{control}-\mathrm{Absorbance}\;\mathrm{of}\;\mathrm{sample}}{\mathrm{Absorbance}\;\mathrm{of}\;\mathrm{control}}\;\times \;100\;\%$$

#### 4.8.4. Ferric Reduction Antioxidant Power (FRAP) Assay 

Reduction of ferric ion (Fe^3+^) to Fe^2+^ by methanolic extract MUSC 125 through the FRAP assay was assessed using the method of Adjimani and Asare [102] with a few alterations. A series of concentrations of methanolic extracts ranging from 5 mg/mL, 10 mg/mL, 20 mg/mL, 40 mg/mL to 80 mg/mL were prepared in 25 µL in 1.5 mL microcentrifuge tubes. This was followed by adding 25 µL of phosphate buffer (0.2 M) and 25 µL from potassium ferricyanide (1%) into sterile 1.5 mL tubes. The reaction mixtures in the microcentrifuge tubes were then heated to 50 °C and temperature kept constant for 20 min before cooling to room temperature. In addition, 25 µL of 10% trichloroacetic acid (TCA) was dispensed into the microcentrifuge tubes to cease the reaction. An 80 µL of the solution was taken out from the microcentrifuge tubes and pipetted into designated wells in a 96 well plate with a further addition of 20 µL of ferric chloride (FeCl_3_). The UV absorbance was determined at the wavelength of 700 nm, and the results expressed in equivalent dose of ascorbic acid. The dose of ascorbic acid equivalents was calculated by first formulating the standard curve (*R*^2^ = 0.97) as indicated below:

(5) $$\mathrm{Ascorbic}\;\mathrm{acid}\;\mathrm{equivalents}\;\left(\mathrm{mg}/\mathrm{mL}\right)=\left(\frac{\mathrm{Absorbance}\;\mathrm{of}\;\mathrm{sample}\;-0.4252}{0.4252}\right)\times \;100\;\%$$

The ascorbic acid equivalents (mg/mL) was then used to determine the actual ascorbic acid dose equivalents using the formula as shown below:

(6) $$\mathrm{Ascorbic}\;\mathrm{acid}\;\mathrm{dose}\;\mathrm{equivalents}\;\left(\mathrm{mg}\right)=\left(\frac{\mathrm{Equation}\;\left(5\right)}{1000}µL\right)\times \;25\;µL$$

### 4.9. TPC by Folin–Ciocalteu’s Reagent Method

The total phenolic content (TPC) of the methanolic extract MUSC 125 was assessed according to Tan, et al. [38]. Several working concentrations of the methanolic extract of MUSC 125 at a volume of 10 µL were transferred accordingly into a 96 well plate and topped with 50 µL of diluted Folin–Ciocalteu’s Reagent (1:10). The 96 well plate was left to stand in dark at room temperature for 5 min. Additionally, 40 µL of sodium carbonate (NaCO_3_) at 7.5% (*w/v*) was dispensed into the wells and permitted to undergo reaction for 30 min. The UV absorbance of the reaction mixture was determined at wavelength of 750 nm and the results presented in terms of gallic acid equivalents. 

### 4.10. Detection of pks and nrps Genes in Strain MUSC 125

Detection of *pks (pks*I and *pks*II) and *nprs* genes in strain MUSC 125 was achieved using the protocol set out by Lee, et al. [88]. In short, degenerate primers consisting of both forward and reverse for *pks*I (K1F-M6R*), pks*II (KSa-KSβ) and *nrps* (A3F-A7R) were employed in the PCR work. The PCR reaction mixture with total volume of 20 µL contained weighed 20 ng–200 ng DNA template, 10 µL of 2× Prime Taq Premix (Genet Bio, Korea), 10 pmoles of primer sets, topped up with sterile ultrapure water. The PCR reaction was performed on Kyratec PCR Supercycler (Kyratec, Queensland, Australia) and the cycling conditions were optimized as follows: (i) 94 °C for 5 min; (ii) 30 cycles of 94 °C for 1 min, 57 °C for 1 min, and 72 °C for 2 min; and (iii) 72 °C for 5 min. After completion of the PCR process, PCR contents were separated under 1.5 % of agarose gel (Promega, USA) and examined through the use Molecular Imager XRS System (Biorad, CA, USA). Gel bands were cut under UV light and purified following the manufacturer’s protocol (GF-1 AmbiClean Kit, Malaysia). Purified DNA sequences in a concentration of ≥30 ng/µL determined by nanodrop were subsequently sequenced. 

### 4.11. Chemical Profiling of Methanolic Extract MUSC 125 with GC-MS

The GC-MS analysis was performed on the methanolic extract MUSC 125 following the method of Tan, et al. [46] which utilized an Agilent Technologies 6980N fitted with 5979 Mass Selective Detector and a HP-5 MS (5% phenyl methyl siloxane) capillary column to carry helium gas at the rate of 1 mLs^−1^. Heat was gradually applied until 40 °C was reached whilst keeping it constant for 10 min; then, it was increased by 3 °C every minute until the peak temperature of 250 °C was reached keeping, it constant for 5 another minutes. The mass spectrometry was functioning at 70 eV. Individual compounds detected by GC-MS were matched with the NIST 05 reference library.

### 4.12. Statistical Analysis

All experiments were in replicates of 3 and the results were quantified as the standard deviation (SD) using the Statistical Package for the Social Sciences software (SPSS). The *p*-value was set at <0.05 using the one-way analysis of variance (ANOVA) and Tukey’s post-hoc test. Pearson’s correlation on total phenolic content and antioxidant assays was also tested.

## 5. Conclusions

The current work well supports the genomic evidence of strain MUSC 125 in an earlier study and establishes the fact that *Streptomyces* sp. MUSC 125 possesses antioxidant potentials in which the methanolic extract of MUSC 125 is effective, not only in chelating ferrous ions but also in scavenging free radicals. On top of that, this study further substantiates the bioactive potentials of strain MUSC 125 with the evidence of promising antibacterial and antibiofilm activities exhibited by the extract. The presence of biosynthetic gene clusters types (PKS I and PKS II) in the genome of strain MUSC 125 and the detection of eight compounds known for various interesting bioactivities, making this strain more attractive for future biotechnological applications. As a whole, this study underscores that Malaysian mangrove soils constitute a valuable source for bioactive *Streptomyces* sp. exhibiting a multitude of biological activities, including anti-MRSA, antibiofilm and antioxidant activities.

## Figures and Tables

**Table 1:** Antioxidant activities of methanolic extract of strain MUSC 125.

Concentration (mg/mL)	Antioxidant Activities (%)		
	ABTS Radical Scavenging Activity	DPPH Radical Scavenging Activity	Metal Chelating Activity
0.125	10.44 ± 2.68 *	3.47 ± 2.49	26.00 ± 3.29 *
0.25	13.32 ± 1.25 *	4.23 ± 1.37 *	28.56 ± 2.62 *
0.5	18.71 ± 1.47 *	9.55 ± 1.01 *	32.49 ± 0.63 *
1	27.27 ± 1.17 *	16.23 ± 3.60 *	39.07 ± 2.61 *
2	41.50 ± 1.06 *	55.00 ± 3.10 *	52.75 ± 1.76 *
4	67.85 ± 2.16 *	64.77 ± 1.31 *	61.10 ± 0.64 *
Gallic Acid ^a^	42.50 ± 0.60 *	-	-
Gallic Acid ^b^	-	53.99 ± 4.06 *	-
EDTA ^c^	-	-	68.49 ± 7.68 *

* Statistically significant at *p* < 0.05. ^a^ Activity of gallic acid at 12.5 μg/mL. ^b^ Activity of gallic acid at 10 μg/mL. ^c^ Activity of Ethylenediaminetetraacetic acid (EDTA) at 0.125 mg/mL.

**Table 2:** GC-MS analysis of the chemical constituents present in the methanolic extract of strain MUSC 125.

Number	Constituents	Retention Time (min)	Molecular Formula	Molecular Weight	Similarity (%)	Biological Activities of Extract	Reference
1	Cyclobutane,2-hexyl-1,1,4-trimethyl	61.569	C_13_H_26_	182	80.4	-	[47]
2	Thiophene, 2-butyl-5-ethyl	61.97	C_10_H_16_S	168	92.5	Antimicrobial	[48]
3	1-Heptyn-3-ol	62.013	C_7_H_12_0	112	95.1	Antifungal, antioxidant	[49]
4	8-[*N*-Aziridylethylamino]-2-6,dimethyloctene-2	62.089	C_14_H_28_N_2_	224	86.7	Antioxidant, antibacterial and anticancer	[50,51]
5	Pyrrolo[1,2-a]pyrazine-1,4-dion,hexahydro	62.815	C_7_H_10_N_2_O_2_	154	95.3	Antioxidant, antimicrobial and algicidal	[44,52]
6	Octahydro-2H-pyrido(1,2-a)pyrimidin-2-one	64.939	C_8_H_14_N_2_O	154	97.7	-	[53]
7	9,9-Dimethyl-3,7-diazabicyclo[3.3.1]nonane	66.304	C_9_H_18_N_2_	154	95.2	Antifungal	[52]
8	2,4-Dihydroxy-6-propylbenzoic acid	66.543	C_10_H_12_O_4_	196	96.1	Antibacterial, antioxidant	[54,55]

- No activity reported. Only the presence of a compound detected by GC-MS.

## Data Availability

The 16SrRNA gene sequence of strain MUSC 125 used to support the findings of this study has been deposited in the GenBank repository with accession number KF682180.

## Supplementary Materials

The following are available online , Table S1: Cultural characteristics of strain MUSC 125, Table S2: Biochemical and physiological characteristics of strain, Table S3: MUSC 125 Anti-MRSA susceptibility test of strain MUSC 125, Table S4: MIC and MBC of methanolic extract of strain MUSC 125, Table S5: Pearson’s correlation coefficients between TPC and antioxidant activities of strain MUSC 125, Figure S1: SEM image capturing the morphology of *Streptomyces* sp. strain MUSC 125, Figure S2. *In-vitro* anti-MRSA of the methanolic extract of strain MUSC 125 activity following agar well diffusion assay measured in terms of zone of inhibition at 10 mg against a) MRSA ATCC 43300 and b) MRSA ATCC 33591, Figure S3: Anti-biofilm/anti-adherence activity of methanolic extract of *Streptomyces* sp. MUSC 125, Figure S4: Ferric reducing activity of methanolic extract of *Streptomyces* sp. strain MUSC 125.
