# Supplementary material for: Streptomyces sp. Strain MUSC 125 from Mangrove Soil in Malaysia with Anti-MRSA, Anti-Biofilm and Antioxidant Activities
Source: Molecules. 2020 Aug 3;25(15):3545. doi: 10.3390/molecules25153545 (PMC7435833; doi:10.3390/molecules25153545)
Supplement: Supplementary file 1 [file molecules-25-03545-s001.pdf]

Supplementary materials

Streptomyces sp. strain MUSC 125 from mangrove soil in Malaysia: Anti-MRSA, anti-biofilm, antioxidant activities and chemical profiling of extract

Table S1. Cultural characteristics of strain MUSC 125

**Media**	**Growth**	**Colony colour**		**Soluble pigment**
		**Aerial mycelia**	**Substrate mycelia**	
ISP2	Well	Yellowish white	Brilliant Yellow	-
ISP3	Poor	Vivid Greenish Yellow	Strong Greenish Yellow	-
ISP4	-	-	-	-
ISP5	Well	Pale Yellowish Green	Pale Yellow	-
ISP6	Well	Greenish White	Yellowish Grey	Dark olive brown
ISP7	Well	Yellowish White	Greenish White	-
AIA	Moderate	Pale Yellowish green	Brilliant Greenish Yellow	-
SCA	Well	Pale Yellowish green	Pale Greenish Yellow	-
SA	Well	Yellowish White	Vivid Yellow	-
NA	Moderate	Greenish White	Greenish Yellow	Greyish greenish yellow

- No growth or nor soluble pigment observed

Table S2. Biochemical and physiological characteristics of strain MUSC 125

Characteristics	Strain MUSC 125
**Biochemical characteristics**	
Catalase	+
Haemolytic	-
**Enzymatic test**	
Chitinase activity (2.5 % chitin)	-
Xylanase activity (0.5 % xylan)	-
Amylolytic activity (0.2 % starch)	+
Protease activity (2 % casein)	-
Lipase activity (1 % tributyrin)	+
Cellulase activity (0.5 % CMC)	-
**Physiological characteristics**	
**Temperature (° C) tolerance**	
Growth	18-40
Optimum	32-36
**NaCl (%) tolerance**	
Growth	0-6
Optimum	0-2
**pH tolerance**	
Growth	4-7
Optimum	7

$^{-}$No activity; $^{+}$Activity

Table S3. Anti-MRSA susceptibility test of strain MUSC 125.

MRSA ATCC strain	Zone of inhibition (mm) at 10 mg/well		
	MUSC 125	Vancomycin (30 μg)	DMSO (0.5 %)
43300	19 ±0	15 ±0	*6 ± 0
33591	19.33 ± 0.58	15.33 ± 0.47	*6± 0

*6 ± 0 is the diameter of the agar well, therefore no activity (N= 3).

Table S4. MIC and MBC of methanolic extract of strain MUSC 125.

MRSA ATCC	MIC (mg/mL)	MBC (mg/mL)	Vancomycin (μg/mL)
43300	12.5	> 50	*5
33591	25	> 50	*10

*MIC of vancomycin.

Table S5. Pearson's correlation coefficients between TPC and antioxidant activities of strain MUSC 125.

Antioxidant activities		
ABTS radical scavenging activity	DPPH radical scavenging activity	Metal chelating activity
r = 0.998*	r = 0.942*	r = 0.974*

*Correlation was significant with a *p* value less than 0.05 level.

Figures

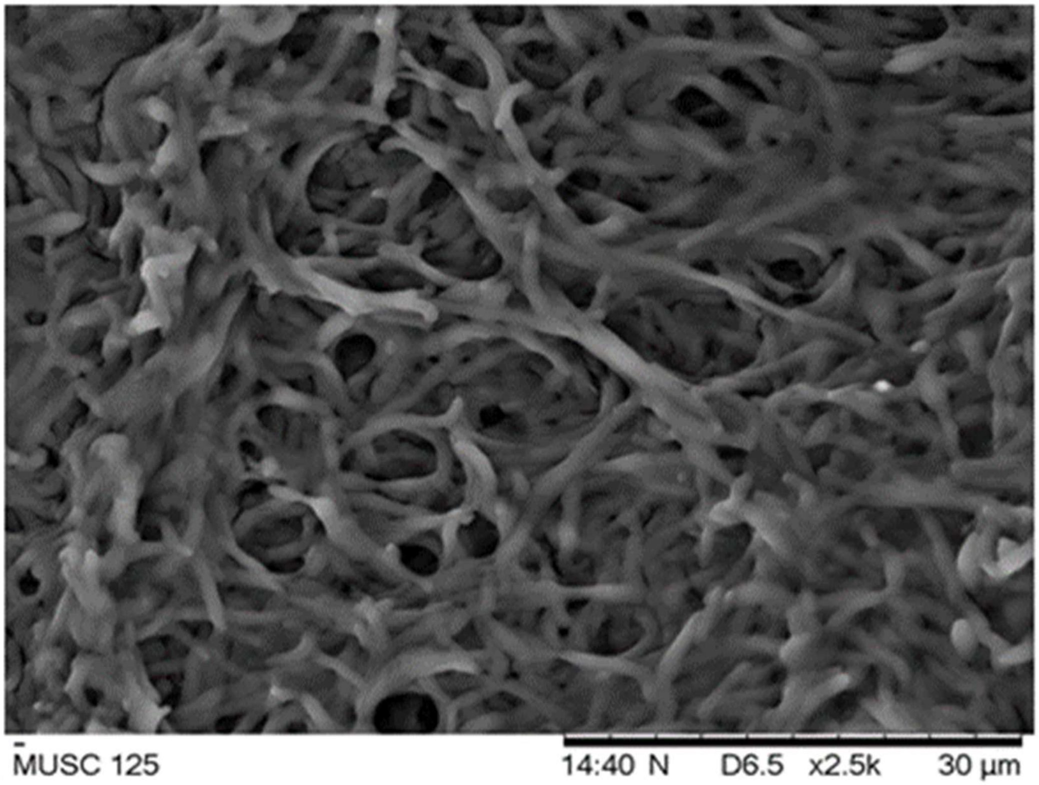

Figure S1. SEM image capturing the morphology of *Streptomyces* sp. strain MUSC 125. This appears to be filamentous with extensive branching, which is a general characteristic of *Streptomyces***.**

A B

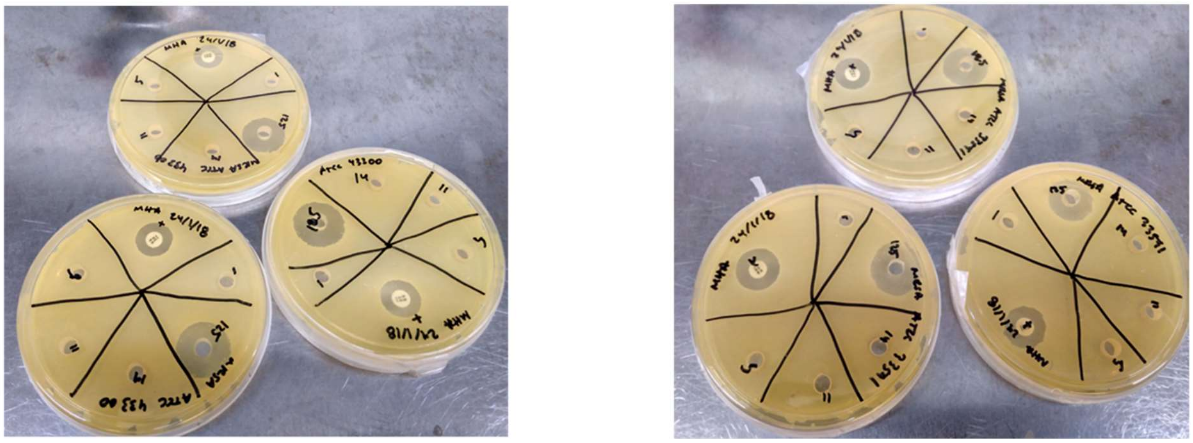

Figure S2. *In-vitro* anti-MRSA of the methanolic extract of strain MUSC 125 activity following agar well diffusion assay measured in terms of zone of inhibition at 10 mg against a) MRSA ATCC 43300 and b) MRSA ATCC 33591. Test done in triplicates.

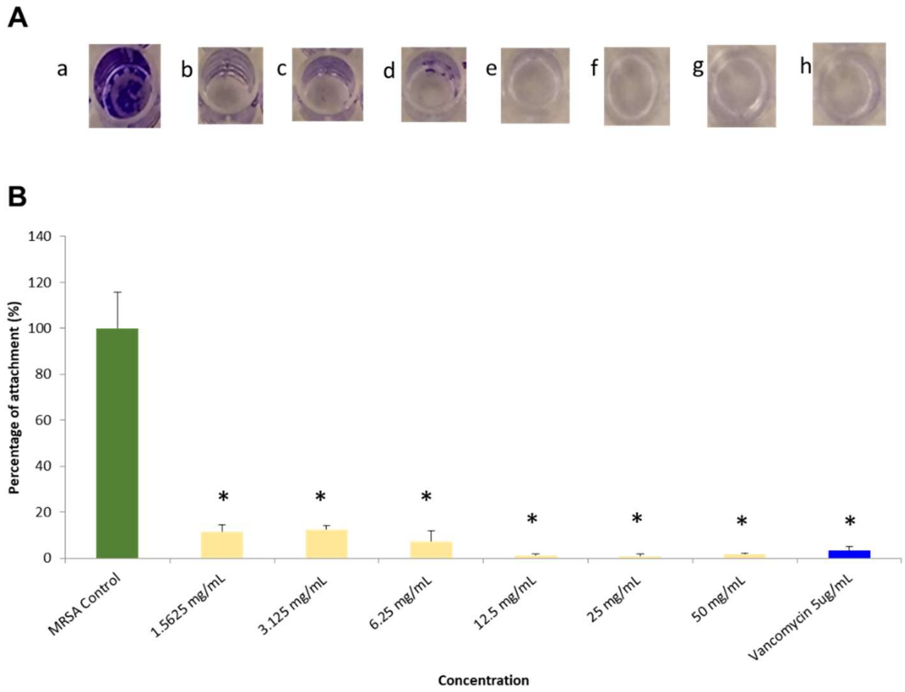

Figure S3. Anti-biofilm/anti-adherence activity of methanolic extract of *Streptomyces* sp. MUSC 125. 3A. Visual observation of effect of methanolic extract at a) Untreated bacteria; b) 1/8× MIC; c)1/4× MIC; d)1/2× MIC; e) 1×MIC; f) 2× MIC; g) 4× MIC; h) Positive control. 3B. Percentage attachment of untreated bacteria, treated bacteria with extract and vancomycin. Data are statistically significant at $p < 0.05$.

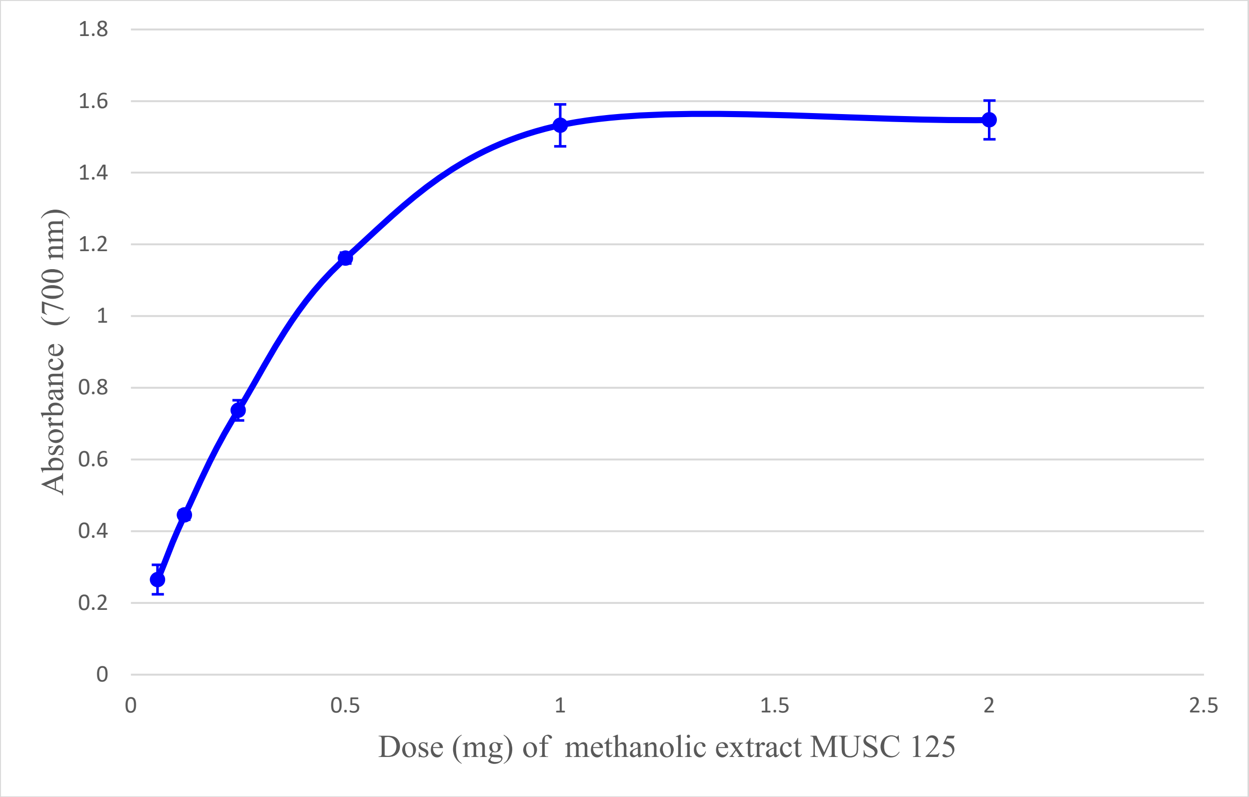

Figure S4. Ferric reducing activity of methanolic extract of *Streptomyces* sp. strain MUSC 125. The 6 doses (0.0625 mg, 0.125 mg, 0.25 mg, 0.5 mg, 1 mg and 2 mg), used in the experiment represent the amount of extract in each of the 6 concentrations (2.5mg/L, 5 mg/mL,10 mg/mL,20 mg/mL, 40 mg/mL and 80 mg/mL) tested present in 25 μL volume. The experiment was run in triplicates (n = 3). Data are statistically significant at $p < 0.05$.
